# KTCNlncDB—a first platform to investigate lncRNAs expressed in human keratoconus and non-keratoconus corneas

**DOI:** 10.1093/database/baw168

**Published:** 2017-01-10

**Authors:** Michał W. Szcześniak, Michal Kabza, Justyna A. Karolak, Malgorzata Rydzanicz, Dorota M. Nowak, Barbara Ginter-Matuszewska, Piotr Polakowski, Rafal Ploski, Jacek P. Szaflik, Marzena Gajecka

**Affiliations:** 1 Department of Genetics and Pharmaceutical Microbiology, Poznan University of Medical Sciences, Poznan, Poland; 2 Department of Integrative Genomics, Institute of Antropology, Adam Mickiewicz University in Poznan, Poznan, Poland; 3 Institute of Human Genetics Polish Academy of Sciences, Poznan, Poland; 4 Department of Medical Genetics, Medical University of Warsaw, Warsaw, Poland and; 5 Department of Ophthalmology, Medical University of Warsaw, Warsaw, Poland

## Abstract

Keratoconus (KTCN, OMIM 148300) is a degenerative eye disorder characterized by progressive stromal thinning that leads to a conical shape of the cornea, resulting in optical aberrations and even loss of visual function. The biochemical background of the disease is poorly understood, which motivated us to perform RNA-Seq experiment, aimed at better characterizing the KTCN transcriptome and identification of long non-coding RNAs (lncRNAs) that might be involved in KTCN etiology. The *in silico* functional studies based on predicted lncRNA:RNA base-pairings led us to recognition of a number of lncRNAs possibly regulating genes with known or plausible links to KTCN. The lncRNA sequences and data regarding their predicted functions in controlling the RNA processing and stability are available for browse, search and download in KTCNlncDB (http://rhesus.amu.edu.pl/KTCNlncDB/), the first online platform devoted to KTCN transcriptome.

**Database URL**: http://rhesus.amu.edu.pl/KTCNlncDB/

## Introduction

Keratoconus (KTCN) is a degenerative disorder associated with stromal thinning and conically shaped protrusion of the cornea, resulting in loss of visual function (1). It is considered as a multifactorial trait with both environmental and genetic etiology, however the exact causes and molecular mechanisms underlying the disease development are not well characterized (2). Importantly, KTCN complexity and heterogeneity make it difficult to recognize the factors triggering KTCN phenotype and we hypothesize that long non-coding RNAs (lncRNAs), defined as transcripts longer than 200 nt that do not code for proteins, might be important players there. A recent survey of lncRNA catalogs showed that there might be >200 000 lncRNA transcripts in human (3), while only a very small fraction of them have been characterized functionally. However, it is well known that they are important players in a number of cellular processes, such as transcription, splicing, translation, protein localization, cell cycle and apoptosis, imprinting, stem cell pluripotency, or cellular structure integrity. They have also been linked to a number of human diseases, in particular cancers (4). This urged us to raise a hypothesis that lncRNAs might also be involved in pathogenesis of KTCN.

**Figure 1.**
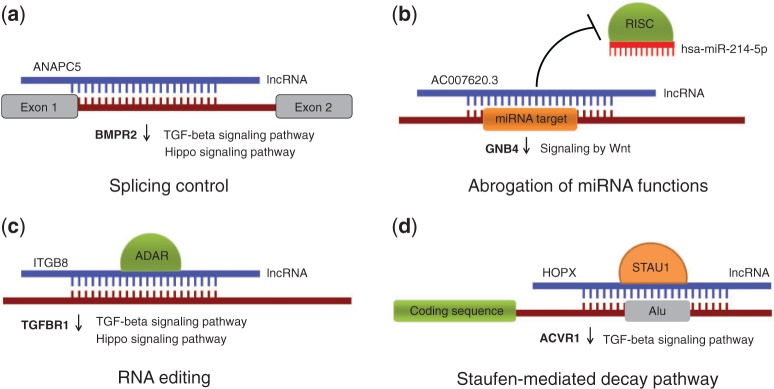
Possible roles of lncRNAs played in the context of RNA-RNA interactions. **(a)** Modulating alternative splicing events through masking splice sites and other splicing signals **(b)** Abrogation of miRNA functions by blocking miRNA target sites; **(c)** Triggering RNA editing by creating dsRNA substrates for ADAR enzymes **(d)** Guiding protein-coding transcripts to degradation in a SMD pathway. Provided are examples of lncRNAs and differentially expressed genes predicted to be under their control. The genes belong to TGF-β, Hippo and Wnt-related signaling pathways and they all are downregulated in KTCN (marked with down arrows).

Here, we performed bioinformatics analysis of RNA-Seq data from KTCN and non-KTCN corneas, including *ab initio* transcriptome assembly, expression estimation, and differential expression analysis and put much emphasis on bioinformatics study of lncRNAs. In order to investigate their potential molecular functions in KTCN, we compiled a list of known and newly identified lncRNAs based on RNA-Seq data from corneas and tried to characterize them functionally, using a previously published methodology (5). In this approach, potential roles of lncRNAs are played in the context of lncRNA-RNA duplexes, with various consequences possible, including (i) splicing modulation by masking splice sites and splicing signals in a pre-mRNA molecule, (ii) triggering mRNA editing events by creating dsRNA structures, (iii) abrogation of miRNA functions by masking their target sites in 3′ untranslated regions (UTRs) and (iv) guiding protein-coding transcripts to degradation within a Staufen-mediated decay (SMD) pathway (Figure 1). There are several experimentally tested lncRNAs that participate in these mechanisms (6–8), while a recent study shows there are >50 000 human transcripts whose metabolism might be affected by formation of lncRNA–RNA duplexes (5). Here, we took all the four possibilities into consideration. The bioinformatics analysis yielded 870 lncRNAs with predicted functions, some of which being able to regulate genes with known or plausible links to KTCN. The list of lncRNAs expressed in human cornea, both from non-KTCN and KTCN samples as well as their potential roles played in the context of RNA–RNA base-pairings are collected in an online database called KTCNlncDB (http://rhesus.amu.edu.pl/KTCNlncDB/). We believe that the data obtained from the first ever use of large-scale RNA-Seq approach of human KTCN cornea samples (Kabza et al., under review), followed by innovative bioinformatics study make KTCNlncDB a unique resource that will be of interest for researchers working in the fields of molecular biology, medicine and biotechnology.

## Materials and Methods

### Sequencing and RNA-seq data analysis

KTCNlncDB was developed based on transcriptomic data from our previous RNA-Seq studies performed for 25 KTCN samples obtained from non-related Polish patients during a keratoplasty procedure and also for 25 non-KTCN corneas, used as controls, collected from patients who were referred for corneal transplantation for different reasons, such as bullous keratopathy, corneal scarring, ulcers, and perforations. Briefly, after RNA extraction and quantitation, libraries were prepared with a TruSeq Stranded Total RNA LT with Ribo-Zero Human/Mouse/Rat Kit (Illumina, San Diego, CA, USA) according to the manufacturer’s protocol, with slight modifications. The performed experimental and in silico procedures have been previously described (Kabza *et al.*, under review). A 75- or 100-bp paired-end run was performed on a HiSeq1500 platform (Illumina). Adapter and trimming of poor quality regions in short reads were performed using Trimmomatic (9). Reads showing similarity to known human rRNAs were excluded from further analysis with Bowtie 2 (10). Short reads were mapped to the human genome (GRCh38) using STAR 2-pass mapping approach (11). Novel transcripts were assembled from mapped reads with StringTie (12) and merged with reference GENCODE 21 annotations using Cuffmerge (13). Novel transcripts were only kept if they had length of at least 200 bp and contained at least one intron. The expression of analyzed genes and transcripts was estimated with Kallisto (14) and differential expression analyses was performed with DESeq2 and edgeR packages (15, 16), using FDR threshold of 0.01 and fold change threshold of 1.5.

### Identification of lncRNAs

Known and newly discovered transcripts associated with differentially expressed genes (from KTCN vs non-KTCN corneas comparison) were used as a starting point. In the search procedure, we followed the commonly accepted criteria (3), so that lncRNA candidates were expected to meet the following requirements: (i) expression of min. 1 TPM in at least one sequencing library; (ii) transcript length of at least 200 nt; (iii) the longest peptide found in the transcript had to be shorter than 100 amino acids (the *translate* method from Biopython was used for *in silico* peptide identification); (iv) transcripts with Ensembl biotypes belonging to ‘Protein coding’ or ‘Short non-coding’ groups were excluded, and in the case of newly discovered transcripts, only those predicted as non-coding by TransDecoder with default settings (17) were kept.

### Identification of lncRNA–RNA interactions

Here, we followed a recently described procedure (5). Briefly, the *lastal* program from the LAST package was used to identify potential lncRNA–RNA interactions (18). A custom substitution matrix was created that enabled a search for G:U (wobble) pairs. In the substitution matrix, G:C, A:T, and G:T matches were scored 4, 2 and 1, respectively; mismatch, gap opening, and gap extension were scored −6, −20 and −8, respectively. A threshold of 500 was applied for alignment scores. In the interaction search procedure, lncRNAs constituted a database, while other transcript sequences were used as a query. The transcripts and human genome annotations were retrieved from Ensembl 81 (19) using BioMart (20) and the download page. When looking for lncRNAs base-pairing with pre-mRNAs, the query transcripts were composed of both exons and introns; any intronic sequences distant by >250 bases from 3′ or 5′ splice sites were masked with *N* characters. With the ‘lastal’ results in MAF format, a set of in-house Python scripts was applied to process the data and prepare them for subsequent steps, which included transforming the coordinates to genomic positions and a MAF-to-BED conversion. The procedure was only carried out for transcripts that showed statistically significant up- or downregulation in the differential gene expression analysis.

### Finding lncRNA–RNA interactions involved in regulatory processes

The lncRNAs, by base-pairing with other RNA molecules, could affect their processing, stability, and expression levels in a number of ways. Therefore, the identified lncRNA–RNA interactions were scanned with the aim of classifying them into the following regulatory mechanisms: modulating alternative splicing through masking splicing signals, abrogation of miRNA functions, triggering RNA editing events, and guiding protein-coding transcripts to degradation in an SMD pathway.

In terms of splicing regulation, interactions between lncRNAs and pre-mRNAs were filtered to keep only those that span exon–intron borders. Then, alternative splice sites were identified based on exon coordinates from Ensembl using in-house Python scripts, and only alignments that overlapped at least one alternative splicing event were kept for further consideration.

Regarding deregulating miRNA functions, Argonaute binding sites obtained from a number of CLIP experiments were downloaded in a BED format from StarBase 2.0 (21). These data, representing putative miRNA binding sites, were put in a single file and converted to non-overlapping coordinates with the ‘merge’ tool from the BEDTools suite (22) to remove data redundancy. Additionally, the coordinates were transformed from hg19 to hg38 genome coordinates using liftOver utility available at UCSC Genome Browser (23). Then, to provide further support for these target sites and to annotate them, *in silico* prediction of mature miRNA target sites was performed with miRanda (24), using default settings. The mature miRNA sequences came from miRBase release 21 (25). The two datasets (miRNA binding sites from StarBase 2.0 and those from the miRanda predictions) were superimposed, and the common part was checked against lncRNA–mRNA alignments. It was required that at least half of the miRNA target site was located within the alignment, yielding miRNA binding sites that are potentially masked by lncRNAs.

In order to find lncRNAs linked to the SMD pathway, coordinates of *Alu* elements in human transcripts were identified with RepeatMasker 4.0.0 (http://www.repeatmasker.org). They were required to be located entirely within the lncRNA–mRNA alignment region and in a 3′ UTR of protein-coding transcripts. Finally, to study lncRNAs in the context of mRNA editing events, known mRNA editing sites were obtained from the RADAR database (26) in a BED format. The coordinates of RNA editing events were transformed from hg19 to hg38 genome coordinates using liftOver utility (23) Then, the lncRNA–mRNA alignments were converted to a BED format with genomic coordinates and checked against downloaded mRNA editing positions. Any adenosine-to-inosine editing event located within an interaction region was considered as potentially triggered by an lncRNA.

### Database construction and testing

The database was constructed using Hypertext Markup Language, cascading style sheets, PHP 5.4 (http://www.php.net/), MySQL 5.5 (http://www.mysql.com/), and Bootstrap 3 framework (http://getbootstrap.com/). The layout was enhanced with JavaScript Highcharts library (http://www.highcharts.com/) for interactive data plotting.

The web interface was tested on Windows (XP, 7, 8, 8.1 and 10) and Mac OS X (Mavericks and Yosemite) operating systems using Internet Explorer (versions 7, 8, 9, 10 and 11), Mozilla Firefox (versions 3.6 and 30), Chrome (version 42), Opera (version 12.15) and Safari (version 8) web browsers. In case of Internet Explorer versions older than 9 the web interface was visualized improperly.

## Results

### Identification of lncRNAs

Using our search criteria, we identified 16 331 lncRNA transcripts, making up 6.38% of the transcriptome. While most of them represent alternative splice forms of known genes, 735 novel lncRNA transcripts belonging to 160 genes were discovered as well. Their functions and possible links to KTCN pathogenesis were investigated, focusing on the putative roles of lncRNAs in the context of RNA–RNA interactions. A total of 56 395 potentially functional base pairings between lncRNAs and differentially expressed genes have been identified, most of which occurring in UTRs of protein-coding genes (91.18%), followed by non-coding transcripts (6.66%) and coding regions (0.99%), and overlapping untranslated and translated regions of protein-coding genes (1.17%). Due to data redundancy, resulting mainly from the fact that a particular lncRNA often base-pairs with multiple splice isoforms of a single gene, these interactions involve 870 unique lncRNAs and 996 transcripts predicted to be under their control. The highest number of lncRNAs has been associated with splicing regulation (789), followed by triggering mRNA editing events (663), masking miRNA target sites (534) and guiding protein-coding transcripts to the SMD pathway (514). The potential functions of 405 (55.10%) novel lncRNAs were also identified, with splicing regulation (403 lncRNAs) being the most prevalent association (Supplementary Table S1).

### A database of lncRNAs

To enable access to the vast lncRNA-associated data generated in this study, we developed KTCNlncDB, a dedicated online database, freely available at http://rhesus.amu.edu.pl/KTCNlncDB/. It contains three main components: (i) ‘lncRNAs page’ with general information concerning all discovered lncRNAs, (ii) ‘lncRNA-RNA interactions page’ with details on functional interactions between lncRNAs and transcripts coming from differentially expressed genes and (iii) ‘BLAST page’, which enables sequence-based search of lncRNAs catalogued in KTCNlncDB.

*lncRNA–RNA interactions page.* This page grants access to lncRNA-RNA interactions data and is divided into three sections, ‘search options’, ‘search summary’ and ‘search results’.

**Search options.** Here, the user can select criteria for data search and filtering: lncRNA id, interacting transcript id, potential function (miRNA deregulation, triggering mRNA editing, guiding to SMD pathway, modulating splicing), gene name, and gene description.

**Search summary.** In this section, user-selected search parameters are provided and the number of found records is reported. In order to download the filtered data into a tab-delimited text file, one needs to press the ‘Download current results’ button. Additionally, a pie chart summarizes lncRNA functions associated with the shown data.

**Search results**. The search results are displayed in a table with one row per lncRNA–RNA interaction. The presented data include IDs of a lncRNA and its mate transcript, predicted regulatory role(s) of the lncRNA, gene name and description, transcript biotype (both referring to a transcript being under lncRNA control) as well as transcript region involved in the interaction (coding sequence, UTR or non-coding sequence). There is also a ‘Details’ button—upon clicking the user gets access to more information on the selected lncRNA–RNA interaction, including functional predictions, expression data and links to other interactions that involve the two RNAs. From this ‘details page’ the user can return to a ‘search page’ without losing search parameters by clicking a ‘Back to search’ button on top of the page.

*lncRNAs page*. This page provides access to all predicted lncRNAs, including those with no functions assigned. Similarly to ‘lncRNA–RNA interactions page’, it is divided into three sections: ‘search options’, ‘search summary’ and ‘search results’ in a form of a table with the following fields: lncRNA transcript and gene ids, genomic coordinates, gene name, transcript biotype and gene description from ENSEMBL. Also, upon clicking ‘Details’ button, more detailed information is displayed: lncRNA sequence in a FASTA format, expression data, best BLAST hits to NONCODE v4 and Swiss-Prot databases as well as *in silico* found short peptides and Ribo-seq data analysis results. The Ribo-seq results come from HEK293 cell line and were provided with a recently published software, RiboTaper (27).

*BLAST page.* Here, one can perform a custom sequence-based search of lncRNAs stored in the database using BLAST version 2.2.26. A user-submitted nucleotide sequence should be in FASTA format. It is possible to select between two tools, BLASTN and MEGABLAST. Except for expectation value (*E*-value) and maximal number of found hits, the search parameters are left default and they are provided for user reference in the same page.

*Download page.* Besides customized downloads available at ‘lncRNA–RNA interactions’ and ‘lncRNAs’ pages, also bulk download is enabled for the lncRNA sequences and their annotations, identified lncRNA–RNA interactions, BLAST search results and peptides found in the lncRNAs.

## Discussion

lncRNAs are believed to be powerful regulators of transcriptomes, yet functionalities of only a small fraction of them have been well established, especially that they outnumber the annotated protein-coding transcripts. Keeping in mind that their involvement in a number of human diseases has already been well documented, we investigated their functions in KTCN, taking advantage of extensive RNA-Seq data from our previous study (Kabza *et al.*, under review). We focused on a scenario wherein lncRNAs play their roles through base pairing with transcripts from differentially expressed genes, based on comparison between KTCN and non-KTCN patients. We found that lncRNAs could regulate processing and expression of at least 996 of them (Supplementary Table S1). This includes a number of genes from the TGF-β, Hippo and Wnt pathways, such as ‘SMAD9’, ‘SMAD6’, ‘TGFB3’ and ‘TGFBR1’, which have already been associated with ocular health (28)*.* We also found a number of KTCN-specific interactions that could account for the observed differences between samples at the transcriptomic level. We did this by keeping only interactions in which both lncRNA and its target transcript are expressed in KTCN but not in non-KTCN samples. We found 1786 such cases, corresponding to 262 distinct genes. These genes show no overrepresentation in Reactome or KEGG pathways (*q*-value < 0.05), as determined with ConsensusPathDB (29), but there are statistically significant results for epidermal growth factor (EGF)-associated pathways in NetPath and Wikipathways. We link this to a recent observation that EGF is upregulated in human uninjured KTCN corneas and significantly reduced in secondary injured KTCN corneas (30). It was proposed that KTCN corneas constantly remain in the injured state but they may be less capable of producing a normal reparative response after a secondary lesion, suggesting that abnormalities in stromal repair are involved in the development of KTCN and may lead to insufficient restoration of the corneal tissue following mechanical trauma, like eye rubbing or contact lens wear (30).

Interestingly, 34.94% of transcripts have more than one lncRNA-mediated regulatory pathway assigned, with ‘SMD + editing’ constituting ∼65% of such cases. We link this to a recently discovered phenomenon that ‘Alus’ function as recruitment elements for the ADAR enzymes that guide adenine-to-inosine editing (31). The same ‘Alus’ are essential for lncRNA–RNA interactions that trigger protein-coding transcript decay within the SMD pathway, thus explaining the SMD-editing association. We also observed that 1329 (29.4%) up- and downregulated genes overlap with other differentially expressed genes. The overlapping loci tend to show the same direction of expression change: in 92.53% of cases, both genes were up- or downregulated. Noteworthy, 578 of 817 overlapping gene pairs contain at least one splice isoform recognized as a lncRNA, reminiscent of results from the GENCODE project (32), where nearly 40% of lncRNAs intersected protein-coding gene loci, with more positive correlations with intersecting mRNAs than expected by chance. These observations are in line with a well-established fact that lncRNAs often regulate gene expression in *cis*, either post-transcriptionally or by the sole act of their transcription, by triggering chromatin modifications such as nucleosomal repositioning, histone modifications, or DNA methylation (33).

The most relevant lncRNA-related results, including predicted lncRNAs and their putative functions in KTCN and non-KTCN transcriptomes, are stored in KTCNlncDB (http://rhesus.amu.edu.pl/KTCNlncDB/). To the best of our knowledge, KTCNlncDB is the first catalogue of lncRNAs and their putative functions in KTCN. As it provides an integrative and easy-to-use platform to investigate lncRNAs expressed in human corneas, we believe KTCNlncDB will significantly contribute to KTCN-related research and also other tasks aimed at deciphering the biology of lncRNAs.

## Supplementary data

Supplementary data are available at *Database* Online.

## Funding

This work was supported by the National Science Centre in Poland (2012/05/E/NZ5/02127 to M.G. and 2014/15/D/NZ2/00525 to M.W.S).

*Conflict of interest*. None declared.
